# An Essential Role of Fyn in the Modulation of Metabotropic Glutamate Receptor 1 in Neurons

**DOI:** 10.1523/ENEURO.0096-17.2017

**Published:** 2017-05-30

**Authors:** Dao-Zhong Jin, Li-Min Mao, John Q. Wang

**Affiliations:** 1 Department of Basic Medical Science, School of Medicine, University of Missouri-Kansas City, Kansas City, Missouri 64108; 2 Department of Anesthesiology, School of Medicine, University of Missouri-Kansas City, Kansas City, Missouri 64108; 3 Beijing Institute of Brain Disorders, Capital Medical University, Beijing 100069, People’s Republic of China

**Keywords:** cerebellum, mGluR, phosphorylation, Src

## Abstract

Fyn is a member of the Src family of nonreceptor tyrosine kinases and is broadly expressed in the CNS. As a synapse-enriched kinase, Fyn interacts with and phosphorylates local substrates to regulate synaptic transmission and plasticity, although our knowledge of specific targets of Fyn at synaptic sites remains incomplete and the accurate role of Fyn in regulating synaptic proteins is poorly understood. In this study, we initiated an effort to explore the interaction of Fyn with a metabotropic glutamate receptor (mGluR). We found that recombinant Fyn directly binds to mGluR1a at a consensus binding motif located in the intracellular C-terminus (CT) of mGluR1a *in vitro*. Similarly, endogenous Fyn interacts with mGluR1a in adult rat cerebellar neurons *in vivo*. Active Fyn phosphorylates mGluR1a at a conserved tyrosine residue in the CT region. In cerebellar neurons and transfected HEK293T cells, the Fyn-mediated tyrosine phosphorylation of mGluR1a is constitutively active and acts to facilitate the surface expression of mGluR1a and to potentiate the mGluR1a postreceptor signaling. These results support mGluR1a to be a novel substrate of Fyn. Fyn, by binding to and phosphorylating mGluR1a, potentiates surface expression and signaling of the receptors.

## Significance Statement

This work identified a novel signaling mechanism in cerebellar neurons. In these neurons, a nonreceptor tyrosine kinase, Fyn, binds to a glutamate receptor [i.e., metabotropic glutamate receptor 1 (mGluR1)] at synaptic sites. This binding enables the kinase to phosphorylate the receptor at a specific tyrosine site, thereby regulating surface expression/trafficking and mGluR1 signaling. These findings unravel a new mechanism underlying the regulation of glutamate receptors by tyrosine kinases in cerebellar neurons and advance the current knowledge on molecular neurobiology of glutamate receptors.

## Introduction

l-Glutamate, a key transmitter in the brain, interacts with ionotropic and metabotropic glutamate receptors (mGluR) to achieve its action (Niswender and Conn, 2010; Traynelis et al., 2010). Among eight mGluR subtypes (mGluR1–8), mGluR1 has been an attractive target in recent studies. As a G-protein-coupled receptor (GPCR), mGluR1 activates G_αq_-coupled phospholipase Cβ1 (PLCβ1). This leads to an increase in phosphoinositide hydrolysis, yielding diacylglycerol and inositol triphosphate (IP_3_) to trigger protein kinase C (PKC) and Ca^2+^ signaling pathways, respectively (Niswender and Conn, 2010). Noticeably, mGluR1 is enriched in the cerebellum (Martin et al., 1992) and is distributed mostly at perisynaptic and postsynaptic sites (Lujan et al., 1996; Kuwajima et al., 2004). Thus, mGluR1 plays a pivotal role in the regulation of synaptic transmission (Traynelis et al., 2010; Nicoletti et al., 2011). mGluR1 is regulated by a phosphorylation-dependent mechanism. Like many other membrane-bound GPCRs, mGluR1 has four intracellular domains, including three intracellular loops (ILs; IL1, IL2, and IL3) and a C-terminal tail. It is the C terminus (CT) that is large in size (359 aa in a long-form splice-variant mGluR1a) and is sufficient to provide a space for protein–protein interactions. In fact, a number of submembranous proteins have been identified to interact with mGluR1 CT (Enz, 2007; 2012; Fagni, 2012). One group of noticeable mGluR1 interacting partners is protein kinases. These kinases are thought to phosphorylate specific residues in mGluR1a CT and thereby modulate function of the modified receptors (Kim et al., 2008; Mao et al., 2011). However, responsible kinases and detailed mechanisms underlying their regulatory roles are poorly understood.

Proteins can be phosphorylated at tyrosine sites by receptor or nonreceptor tyrosine kinases. Src family kinases (SFKs), a subfamily of nonreceptor tyrosine kinases (Neet and Hunter, 1996), have been studied mostly for their roles in phosphorylating and regulating proteins. Five SFK members of a total of nine are expressed in the brain (Mao and Wang, 2016). Among these five SFK members, Fyn (isoform 1, also known as FynB), a 59 kDa protein, is of particular interest (Cooke and Perlmutter, 1989; Saito et al., 2010). This kinase is enriched at synaptic structures. Thus, Fyn is thought to function at synaptic sites and act as a key regulator in synaptic transmission and plasticity (Neet and Hunter, 1996). Indeed, Fyn tyrosine-phosphorylates ionotropic glutamate receptors and other synaptic proteins, thereby modulating the expression and function of these receptors/proteins and synaptic signaling (Ohnishi et al., 2011; Schenone et al., 2011). However, at present, whether and how Fyn regulates mGluRs remain elusive.

This study therefore explored possible Fyn–mGluR interactions. We found that mGluR1a is a novel substrate of Fyn. Recombinant Fyn directly binds to mGluR1a at its CT region *in vitro*. Endogenous Fyn forms complexes with mGluR1a in rat cerebellar neurons *in vivo*. Active Fyn phosphorylates the mGluR1a CT at a specific tyrosine site. This phosphorylation is functionally relevant as it significantly modulates surface expression and mGluR1a signaling in cerebellar neurons and HEK293T cells. Together, we have discovered a previously unrecognized Fyn–mGluR1a coupling that is involved in the regulation of mGluR1a.

## Materials and Methods

### Animals

Adult male Wistar rats (weight, 200–300 g) were purchased from Charles River. Animals were individually housed at a temperature of 23°C and a humidity of 50 ± 10% with food and water available *ad libitum*. The animal room was on a 12 h light/dark cycle with lights on at 7:00 A.M. All animal use procedures were in strict accordance with the National Institutes of Health *Guide for the Care and Use of Laboratory Animals* and were approved by the Institutional Animal Care and Use Committee.

### Glutathione *S*-transferase fusion protein synthesis

Glutathione *S*-transferase (GST) fusion proteins were synthesized. The cDNA fragments encode mGluR1a-CT1(K841-T1000), mGluR1a-CT2(P1001-L1199), mGluR1a-CT1a(K841-N885), mGluR1a-CT1b(A886-K931), mGluR1a-CT1c(N925-T1000), mGluR1a-IL1(R618-E629), mGluR1a-IL2(K678-Q706), mGluR1a-IL3(K773-K785), GluA1-CT (E809-L889), and GluA2-CT (E834-I883). These cDNA fragments were generated by PCR amplification from full-length (FL) cDNA clones (rat mGluR1; UniProtKB accession #P23385) and subcloned into BamHI-EcoRI sites of the pGEX4T-3 plasmid (GE Healthcare). To confirm appropriate splice fusion, constructs were sequenced. GST fusion proteins were expressed in *Escherichia coli* BL21 cells and purified as described by the manufacturer.

### Cell cultures and transfection

HEK293T cells were cultured in Eagle’s Minimum Essential Medium from American Type Culture Collection at 37°C with 5% CO_2_. The medium contains 10% fetal bovine serum and 1% penicillin-streptomycin (Sigma-Aldrich). Transfections were conducted on an ∼70–80% confluent monolayer using Lipofectamine 2000 (Thermo Fisher Scientific) according to the manufacturer instructions. Experiments were performed 18 h after the transfection. Mammalian expression vectors for expressing proteins with a FLAG tag include FL rat mGluR1a and Fyn wild type (WT) in pcDNA3.1 + C(K)DYK vectors bearing the CMV promoter (GenScript) and the pcDNA3.1 + C(K)DYK empty vector control. Site-directed mutations were introduced into pcDNA3.1(+) constructs containing mGluR1a or Fyn by a QuikChange site-directed mutagenesis kit (Stratagene).

### Affinity purification (pull-down) assay

Pull-down assays were conducted with solubilized rat cerebellar lysates (50–100 μg) according to the procedures described previously (Liu et al., 2009; Guo et al., 2010). At least three experiments were performed for each analysis.

### *In vitro* binding assay

Recombinant His-tagged active Fyn (FynB) with an FL of 537 aa (10 ng; Millipore), His-tagged paxillin (10 ng; RayBiotech), FLAG-tagged focal adhesion kinase (Fak; 10 ng) or FLAG-tagged Fyn mutant (Y531F or K299M) was equilibrated to binding buffer containing 200 mm NaCl, 0.2% Triton X-100, 0.1 mg/ml bovine serum albumin (BSA), and 50 mm Tris, pH 7.5. Binding reactions were initiated by adding purified GST fusion proteins and continued for 2–3 h at 4°C. We then used glutathione Sepharose 4B beads (10%, 100 μl) to precipitate GST fusion proteins. After the precipitate was washed three times, bound proteins were eluted with 4× lithium dodecyl sulfate (LDS) loading buffer, resolved by SDS-PAGE, and immunoblotted with the antibodies indicated.

### Phosphorylation assays *in vitro*

GST fusion proteins or His-tagged paxillin (0.1–0.5 μm) were incubated with recombinant active Fyn (Sigma-Aldrich) or Fak (Creative Biomart) for 30 min (30°C) in a reaction buffer (25 μl) containing 10 mm HEPES, pH 7.4, 10 mm MgCl_2_, 1 mm Na_3_VO_4_, 1 mm dithiothreitol, 0.1 mg/ml BSA, and 50 μm ATP. An amount of 2.5 μCi/tube [γ-^32^P]ATP (∼3000 Ci/mmol; PerkinElmer) was added for autoradiography. Phosphorylation reactions were stopped by adding and boiling the LDS sample buffer (3 min). Phosphorylated proteins were resolved by SDS-PAGE and visualized by autoradiography or immunoblotting. The amount of radioactivity that was incorporated into the substrate bands was assessed by liquid scintillation counting of the radioactive substrate bands excised from the gels. At least three experiments were performed for each analysis. Phosphorylation stoichiometry was determined by the amount of radioactive phosphate incorporated into substrate (mol phosphate/mol substrate).

### Dephosphorylation reactions

GST fusion proteins that were phosphorylated by the above procedure were precipitated, and the supernatant containing Fyn was removed. Precipitates were washed twice and were suspended in a solution containing 50 mm Tris-HCl, pH 8.5, 1 mm MgCl_2_, 0.1 mm ZnCl_2_, and calf intestine alkaline phosphatase (CIP; 100 units/ml; Roche). The suspension was incubated for 1 h at 37°C. Dephosphorylation reactions were stopped by adding an LDS sample buffer. Samples were then subjected to standard gel electrophoresis and immunoblotting.

### Coimmunoprecipitation

The rat cerebellum was removed after anesthesia and decapitation and was homogenized in cold isotonic homogenization buffer containing 0.32 m sucrose, 10 mm HEPES, pH 7.4, 2 mm EDTA, and a protease/phosphatase inhibitor cocktail (Thermo Fisher Scientific). Lysates were then processed at 4°C. After centrifugation (800 × *g*, 10 min), the supernatant was centrifuged again (10,000 × *g*, 15 min) to get P2 synaptosomal pellets. P2 pellets were solubilized in the buffer containing Triton X-100 (0.5%, v/v), 1% sodium deoxycholate, and a protease/phosphatase inhibitor cocktail for 1 h. After centrifugation, the solubilized supernatant was used for coimmunoprecipitation. HEK293T cells were lysed and solubilized in RIPA buffer (Sigma-Aldrich). Solubilized supernatant proteins after centrifugation were used for coimmunoprecipitation. Solubilized samples were incubated with a rabbit or mouse antibody. The complex was precipitated with 50% protein A and G agarose/Sepharose bead slurry (GE Healthcare). Proteins were separated on Novex 4–12% gels and probed with immunoblotting with a mouse or rabbit antibody if a rabbit or mouse antibody was used in immunoprecipitation, respectively.

### Cerebellar slice preparation

After rats were anesthetized and decapitated, rat brains were removed. The cerebellum was cut into coronal slices (300 μm) using a VT1200S vibratome (Leica). Slices were preincubated in artificial CSF (ACSF) containing the following (in mm): 10 glucose, 124 NaCl, 3 KCl, 1.25 KH_2_PO_4_, 26 NaHCO_3_, 2 MgSO_4_, and 2 CaCl_2_, bubbled with 95% O_2_/5% CO_2_, pH 7.4, in an incubation tube at 30°C under constant oxygenation with 95% O_2_/5% CO_2_ for 60 min. Additional preincubation after the solution was replaced with fresh ACSF was made for 10–20 min. Drugs were added and incubated at 30°C. Slices were collected after drug treatment for neurochemical assays.

### Surface protein biotinylation

Surface protein biotinylation on brain slices was performed following established protocols (Kato et al., 2012; Knackstedt et al., 2013; Pabba et al., 2014). Briefly, rat cerebellar slices (300 μm) after drug treatments were incubated in ACSF containing 1 mg/ml EZ-LINK-Sulfo-NHS-SS-Biotin (Thermo Fisher Scientific) for 45 min at 4°C. To biotinylate surface proteins of HEK293T cells, cells were incubated with EZ-LINK-Sulfo-NHS-SS-Biotin for 30 min at 4°C. Unreacted biotinylation reagent was removed by washing and was quenched by 100 mm glycine. Slices and HEK293T cells were homogenized by sonication in an HEPES-Triton-SDS lysis buffer containing the following (in mm): 25 HEPES, 150 NaCl, 1% Triton X-100, 0.5% SDS, and a protease/phosphatase inhibitor cocktail (Thermo Fisher Scientific). After centrifugation (1000 × *g*, 10 min) at 4°C, the supernatant was collected and used as the total protein fraction. An equal aliquot of total proteins was incubated with neutrAvidin resin (Thermo Fisher Scientific) overnight at 4°C. Biotinylated proteins (i.e., surface proteins) were precipitated by centrifugation and were then eluted with an LDS sample buffer (boiling for 3 min). The abundance of proteins of interest in surface and total fractions was analyzed by immunoblotting.

### Western blot

To separate proteins, SDS NuPAGE Bis-Tris 4–12% gels (Invitrogen) were used. Separated proteins were transferred to polyvinylidene fluoride membranes. We then incubated membranes with primary antibodies overnight at 4°C, which was followed by incubation with secondary antibodies. An enhanced chemiluminescence reagent (GE Healthcare) was used to develop immunoblots.

### IP_3_ assays

Intracellular IP_3_ levels in rat cerebellar slices or HEK293T cells were measured using a HitHunter IP_3_ Fluorescence Polarization Assay Kit from DiscoveRx or a rat IP3 ELISA kit from CUSABIO following the manufacturer instructions (Jin et al., 2013; Cansev et al., 2015; Tabatadze et al., 2015).

### Antibodies and pharmacological agents

The antibodies we used in this study include rabbit antibodies against mGluR1a (Millipore), Src with phosphorylated tyrosine 416 (pan-pY416, Cell Signaling Technology), Fyn (Cell Signaling Technology), Src (Cell Signaling Technology), phosphotyrosine (pY; Millipore), Fak (Cell Signaling Technology), paxillin (Cell Signaling Technology), FLAG (Cell Signaling Technology), or β-actin (Sigma-Aldrich), or mouse antibodies against mGluR1a (BD), Fyn (Santa Cruz Biotechnology), pY (PY20, BD), or transferrin receptors (TfRs; Thermo Fisher Scientific). The antibody against pY416 reacts with the following Src family members when autophosphorylated at a conserved activation residue: Y416 (chicken Src), Y419 (rat Src), and Y420 (rat Fyn). Pharmacological agents, including (*S*)-3,5-dihydroxyphenylglycine (DHPG), 3-methyl-aminothiophene dicarboxylic acid (3-MATIDA), 3-(4-chlorophenyl) 1-(1,1-dimethylethyl)-1*H*-pyrazolo[3,4-*d*]pyrimidin-4-amine (PP2), 1-phenyl-1*H*-pyrazolo[3,4-*d*]pyrimidin-4-amine (PP3), and oxotremorine-M, were purchased from Sigma-Aldrich. All drugs were freshly prepared on the day of the experiments.

### Statistics

The results in this study are presented as the mean ± SEM. Data were evaluated using the Student’s *t* test or a one-way ANOVA followed by a Bonferroni (Dunn) comparison of groups using least-squares-adjusted means. Probability levels of <0.05 were considered to be statistically significant.

## Results

### Phosphorylation of mGluR1a by Fyn

**Figure 1.**
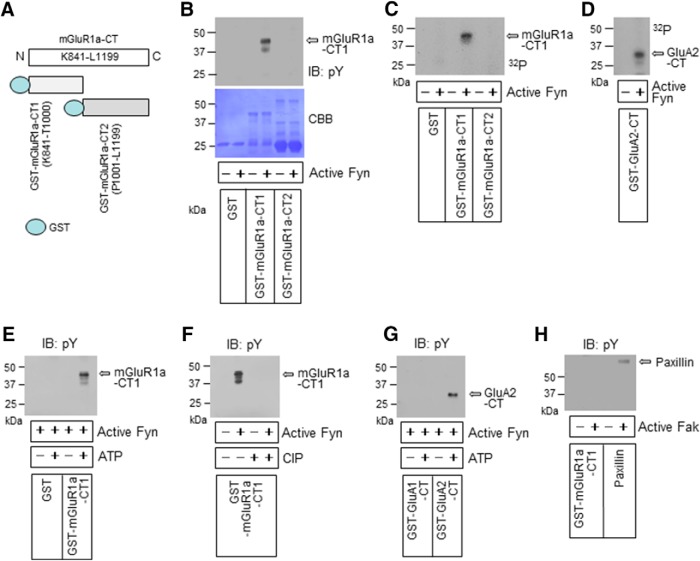
Phosphorylation of mGluR1a by Fyn. ***A***, GST-mGluR1a-CT1 and GST-mGluR1a-CT2 proteins synthesized from rat mGluR1a-CT FL. ***B***, A representative immunoblot illustrating phosphorylation of GST-mGluR1a-CT1 by Fyn. Tyrosine phosphorylation levels were detected by an antibody against pY. A membrane staining with Coomassie Brilliant Blue (CBB) is present below to show protein loading. Note that GST-mGluR1a-CT1 was phosphorylated, while GST-mGluR1a-CT2 and GST alone were not. ***C***, A representative autoradiograph illustrating phosphorylation of GST-mGluR1a-CT1 by Fyn. ***D***, An autoradiograph illustrating phosphorylation of GST-GluA2-CT by Fyn. ***E***, Phosphorylation of GST-mGluR1a-CT1 in the presence but not the absence of ATP. ***F***, Dephosphorylation of Fyn-mediated phosphorylation of GST-mGluR1a-CT1 by CIP. ***G***, Phosphorylation of GST-GluA2-CT by Fyn. In contrast to GST-GluA2-CT, phosphorylation of GST-GluA1-CT was not observed. ***H***, A representative immunoblot showing phosphorylation responses of GST-mGluR1a-CT1 to Fak. Note that Fak did not phosphorylate mGluR1a-CT1, while it phosphorylated paxillin, a known substrate of Fak. All phosphorylation reactions were conducted with active Fyn or Fak at 30°C for 30 min (***B–H***) followed by dephosphorylation reactions (***F***). The reactions were subjected to gel electrophoresis followed by autoradiography (***C***, ***D***) or immunoblot (IB) with an anti-pY antibody (***B***, ***E–H***). Open arrows indicate phosphorylated GST-mGluR1a-CT1 (***B***, ***C***, ***E***, ***F***), GST-GluA2-CT (***D***, ***G***), or paxillin (***H***). N, NH_2_-terminus; C, COOH-terminus.

Intracellular domains of mGluR1a include IL1, IL2, IL3, and CT. Notably, only the CT region contains tyrosine residues. To explore possible phosphorylation at any of these tyrosine residues, we synthesized two GST fusion recombinant proteins covering the different segments of CT [i.e., mGluR1a-CT1(K841-T1000) and mGluR1a-CT2(P1001-L1199)] in addition to a GST protein (Fig. 1*A*). These three proteins were then used for testing their tyrosine phosphorylation in response to active Fyn in phosphorylation assays *in vitro*. Using an anti-phosphotyrosine antibody, we found phosphorylation signals in GST-mGluR1a-CT1 but not GST-mGluR1a-CT2 and GST alone (Fig. 1*B*). Similarly, through monitoring the incorporation rate of ^32^P into the proteins, we detected phosphorylation in CT1 but not CT2 (Fig. 1*C*). Stoichiometric ratios of 0.48 ± 0.02 and 0.89 ± 0.05 mol phosphate/mol CT1 were obtained after 2 and 30 min of incubation, respectively. Phosphorylation was also seen in GST-GluA2-CT (Fig. 1*D*). Since the AMPA receptor GluA2 subunit is a known substrate of Fyn (Hayashi and Huganir, 2004), this phosphorylation response of GluA2-CT served as a positive control. These results reveal that mGluR1a is among the proteins subjected to tyrosine phosphorylation by Fyn. Consistent with this notion, Fyn failed to induce phosphorylation of CT1 in the absence of the phosphate donor ATP (Fig. 1*E*). The Fyn-induced phosphorylation of CT1 was dephosphorylated by a serine/threonine and tyrosine dual-specificity phosphatase CIP (Edbauer et al., 2009; Orlando et al., 2009; Fig. 1*F*). In addition, active Fyn phosphorylated GluA2-CT but not GluA1-CT of AMPA glutamate receptors (Fig. 1*G*), which is similar to the observations in a previous study (Hayashi and Huganir, 2004). Unlike Fyn, another nonreceptor tyrosine kinase Fak did not phosphorylate mGluR1a-CT1, while it phosphorylated its own known substrate paxillin (Panetti, 2002; Fig. 1*H*). Together, the results obtained above reveal mGluR1a as a new substrate of Fyn.

**Figure 2.**
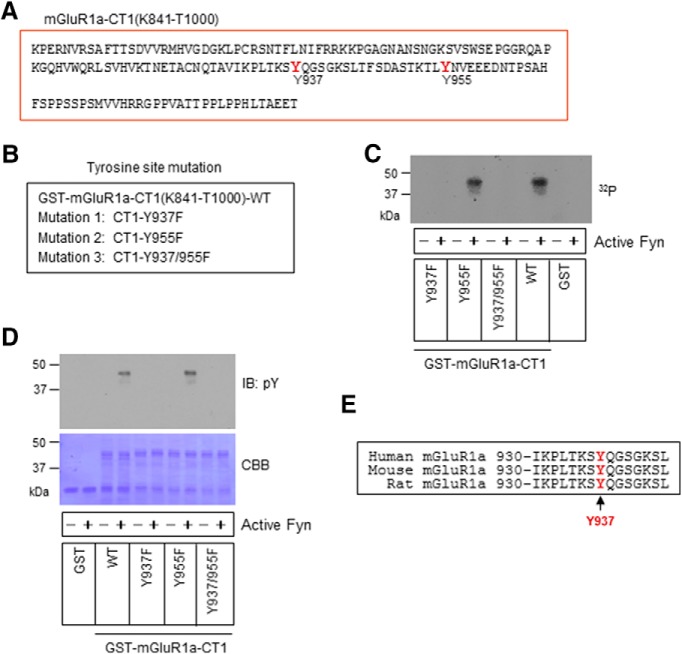
Phosphorylation of a tyrosine residue in mGluR1a-CT1 by Fyn. ***A***, Amino acid sequence of mGluR1a-CT1(K841-T1000). Two tyrosine residues, Y937 and Y955 (in red), are contained in this fragment. ***B***, Site-directed mutation proteins derived from mGluR1a-CT1. Mutants include mutations of Y937 to phenylalanine (Y937F), Y955 to phenylalanine (Y955F), and both tyrosine sites (Y937 and Y955) to phenylalanine (Y937/955F). ***C***, A representative autoradiograph showing phosphorylation of GST-mGluR1a-CT1 mutants and WT induced by active Fyn. Note that the Y955F mutant and WT showed similar phosphorylation by Fyn, while the Y937F and Y937/955F mutants showed no phosphorylation. ***D***, A representative immunoblot showing phosphorylation responses of the three mutants to active Fyn. Again, only the Y955F mutant and WT exhibited phosphorylation signals. A membrane staining with Coomassie Brilliant Blue (CBB) is present below to show protein loading. ***E***, The conserved Y937 residue (in red) in human, rat, and mouse mGluR1a. Phosphorylation of mGluR1a-CT1 (mutants or WT) was detected by ^32^P incorporation autoradiography (C) or immunoblot (IB) with an antibody against pY (D).

There are two tyrosine sites in mGluR1a-CT1: tyrosine 937 (Y937) and tyrosine 955 (Y955; Fig. 2*A*). We next tested the phosphorylation response of these two sites to Fyn to identify the accurate phosphorylation site. To this end, we synthesized CT1 proteins carrying site-directed mutations (Fig. 2*B*). We then compared these mutants with WT CT1 in phosphorylation reactions. Noticeably, the mutation of Y937 to phenylalanine (mutation 1, Y937F) abolished CT1 phosphorylation as detected by ^32^P incorporation (Fig. 2*C*). In contrast, the mutation of Y955 (mutation 2, Y955F) did not affect CT1 phosphorylation. No phosphorylation was detected in the mutant bearing mutations in both sites (mutation 3, Y937/955F). Similar results were observed in assays in which phosphorylation responses were detected by an anti-pY antibody (Fig. 2*D*). Evidently, Y937 is a primary site subjected to phosphorylation by Fyn, while Y955 is insensitive to Fyn. Amino acid alignment analysis reveals conservation of Y937 in the human, rat, and mouse mGluR1a (Fig. 2E).

### Binding activity between Fyn and mGluR1a

**Figure 3.**
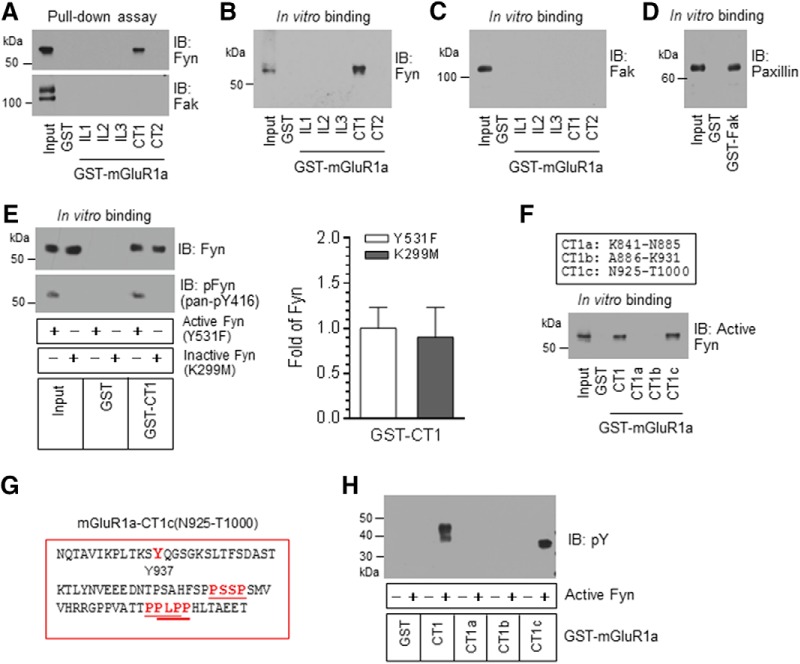
Interactions between Fyn and mGluR1a. ***A***, Pull-down assays with immobilized GST fusion proteins containing distinct intracellular domains of mGluR1a. GST fusion proteins were incubated with adult rat cerebellar lysates. Note that CT1 but not other intracellular domains precipitated endogenous Fyn. No GST fusion proteins precipitated Fak. ***B***, ***C***, *In vitro* binding assays with immobilized GST fusion proteins and purified active Fyn (***B***) and Fak (***C***). Note that CT1 precipitated Fyn (***B***) but not Fak (***C***). ***D***, *In vitro* binding assays showing that GST-Fak precipitated paxillin. ***E***, Comparison between active and inactive Fyn in binding to mGluR1a-CT1. A constitutively active Fyn mutant (Y531F) and a kinase-dead, inactive Fyn mutant (K299M) were used. Representative immunoblots are shown to the left of the quantified data. ***F***, *In vitro* binding assays with truncated mGluR1a-CT1 fragments (CT1a-c). Note that CT1c but not CT1a and CT1b precipitated Fyn. ***G***, Fyn SH3 binding motifs (PXXP) in the CT1c region. Three PXXP motifs (underlined, in red) are revealed. ***H***, Tyrosine phosphorylation of CT1c by Fyn. Endogenous and recombinant Fyn proteins bound to GST fusion proteins were visualized by immunoblot (IB). Tyrosine phosphorylation of CT1c was detected by an anti-pY antibody. Data are presented as the mean ± SEM (*n* = 5 per group).

Fyn binds to many of its known substrates (Knox and Jiang, 2015). To determine whether Fyn binds to mGluR1a, we conducted a series of protein–protein binding assays. In these assays, we used GST fusion proteins containing discrete intracellular domains of mGluR1a as immobilized molecules to precipitate a target binding partner [i.e., Fyn]. In pull-down assays, GST-mGluR1a-CT1 precipitated native Fyn from adult rat cerebellar lysates, while GST alone and other GST fusion proteins containing IL1, IL2, IL3, and CT2 did not (Fig. 3*A*). No GST fusion proteins precipitated Fak (Fig. 3*A*). Fyn is predominantly expressed in the cerebellum (Umemori et al., 1992; Bare et al., 1993), and meanwhile Fak is also expressed in this region (Burgaya et al., 1995; Menegon et al., 1999). To determine whether Fyn directly binds to mGluR1a, we performed *in vitro* binding assays with purified Fyn and mGluR1a proteins. GST-mGluR1a-CT1 bound to and precipitated Fyn (Fig. 3*B*). GST alone and other GST fusion proteins showed no precipitation of Fyn. Fak was not precipitated by any GST fusion proteins (Fig. 3*C*). GST-Fak precipitated its known substrate paxillin (Fig. 3*D*). These results support that Fyn binds to mGluR1a at the CT1 region of the receptor.

We next compared active and inactive Fyn for their binding activity to mGluR1a-CT1. As shown in Figure 3*E*, a constitutively active form of Fyn (i.e., Fyn-Y531F; Cheng et al., 1991), bound to CT1. So did an inactive form of Fyn [i.e., a kinase-dead and dominant-negative mutant of Fyn (K299M); Twamley et al., 1992; Twamley-Stein et al., 1993]. The inactive form of K299M was confirmed by the lack of phosphorylation at a conserved and activation-associated autophosphorylation site (Y416; Roskoski, 2005; Okada, 2012). To further narrow down the binding region, we tested different parts of CT1 in their binding activity to Fyn. CT1c (N925-T1000) precipitated Fyn (Fig. 3*F*). In contrast, CT1a (K841-N885) and CT1b (A886-K931) did not. Thus, CT1c is a core region harboring Fyn. Interestingly, the Fyn-mediated phosphorylation site Y937 resides within this region. Adjacent to Y937, there are three consensus proline-rich motifs (PXXP, where “X” represents any amino acid; Fig. 3*G*) where Fyn could bind via its Src-homology 3 (SH3) domain (Ren et al., 1993; Hauck et al., 2001), a highly conserved platform among SFKs for protein–protein interactions (Saito et al., 2010). As expected, CT1c, but not CT1a and CT1b fragments, was tyrosine phosphorylated by Fyn (Fig. 3*H*).

### Interactions of Fyn with mGluR1a in cerebellar neurons

**Figure 4.**
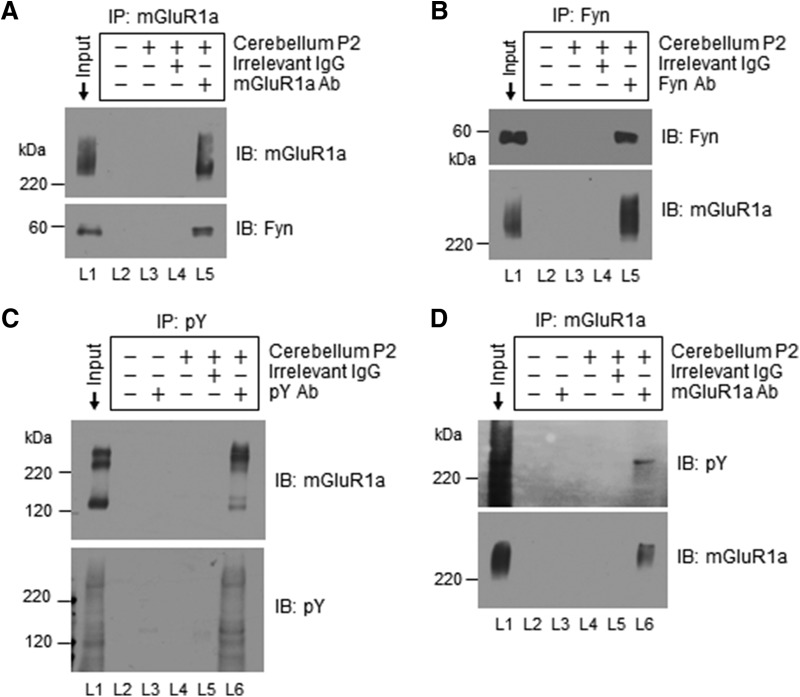
Coimmunoprecipitation of Fyn with mGluR1a and tyrosine phosphorylation of mGluR1a in rat cerebellar neurons. ***A***, Coimmunoprecipitation (IP) of Fyn and mGluR1a with an anti-mGluR1a antibody (Ab). ***B***, Reverse coimmunoprecipitation of Fyn and mGluR1a with an anti-Fyn antibody. Note that Fyn and mGluR1a were seen in mGluR1a and Fyn precipitates, respectively (lane 5). No specific bands were seen in lanes 3 and 4 due to the lack of a precipitating antibody (L3) and the use of an irrelevant IgG (L4). ***C***, Tyrosine-phosphorylated mGluR1a detected in immunoprecipitated pY proteins. Phosphotyrosine proteins were precipitated by an anti-pY antibody. mGluR1a was clearly seen in pY precipitates. ***D***, Tyrosine phosphorylation of mGluR1a detected in immunoprecipitated mGluR1a proteins. Solubilized synaptosomal proteins (P2) from the cerebellum were used in IP assays. Precipitated proteins were visualized by immunoblots (IB) with indicated antibodies.

Our next attempt was to determine whether Fyn interacts with mGluR1a in neurons. This was examined in coimmunoprecipitation assays using the rat cerebellar tissue because mGluR1 but not mGluR5 is expressed in the cerebellum (Martin et al., 1992; Shigemoto et al., 1993) and Purkinje neurons, the principal neurons of cerebellar cortex, express mGluR1a as a predominant splice variant (Fotuhi et al., 1993). In a coimmunoprecipitation assay in which an anti-mGluR1a antibody was added into solubilized synaptosomal protein samples (P2), we observed strong mGluR1a immunoreactivity (Fig. 4*A*). Noticeably, Fyn immunoreactivity was also exhibited in mGluR1a precipitates, indicating that endogenous Fyn and mGluR1a form complexes in cerebellar neurons *in vivo*. This notion is supported by a reverse coimmunoprecipitation assay in which an anti-Fyn antibody was added to precipitate Fyn. mGluR1a immunoreactivity was seen in Fyn precipitates (Fig. 4*B*). To determine whether native mGluR1a in cerebellar neurons is tyrosine phosphorylated, we immunoprecipitated pY proteins from the cerebellum using an anti-pY antibody. In this defined pool of pY proteins, we found a detectable amount of mGluR1a (Fig. 4*C*). In immunoprecipitated mGluR1a proteins, we also saw pY signals (Fig. 4*D*). Thus, mGluR1a in cerebellar neurons is among a subset of proteins subjected to tyrosine phosphorylation under normal conditions.

### Phosphorylation of cerebellar mGluR1a by SFKs

**Figure 5.**
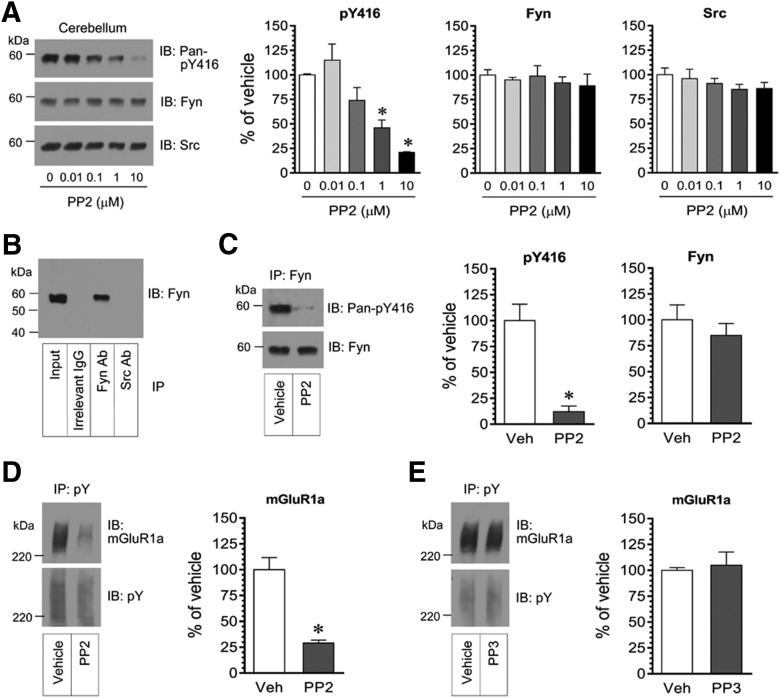
Tyrosine phosphorylation of mGluR1a in rat cerebellar neurons. ***A***, Effects of PP2 on Y416 phosphorylation and expression of Fyn and Src in the cerebellum. Note that PP2 concentration-dependently reduced pan-pY416 levels, while PP2 did not affect Fyn and Src expression. ***B***, Immunoprecipitation of Fyn from the cerebellum. Cerebellar Fyn was immunopurified by a Fyn antibody but not by an Src antibody or irrelevant IgG. ***C***, Effects of PP2 on Y416 phosphorylation of Fyn proteins immunopurified from the cerebellum. Note that Fyn Y416 phosphorylation was almost abolished by PP2. ***D***, ***E***, Effects of PP2 (***D***) and PP3 (***E***) on tyrosine phosphorylation of mGluR1a. Note that the amount of tyrosine-phosphorylated mGluR1a was drastically reduced by PP2 but not PP3. Tyrosine phosphorylation of mGluR1a was detected by immunoprecipitation of pY proteins, followed by immunoblot analysis of mGluR1a in pY precipitates. Representative immunoblots are shown to the left of the quantified data (***A***, ***C–E***). Rat cerebellar slices were incubated with vehicle (Veh), PP2, or PP3 at the range of concentrations indicated (***A***) or at 10 μm (***C–E***) for 30 min. Slices were then collected for immunoblots (IB; ***A***) or immunoprecipitation (IP) followed by immunoblots (***B–E***). Data are presented as means ± SEM (*n* = 3-5 per group) and were analyzed by one-way ANOVA (***A***) or Student’s *t* test (***C–E***). *p < 0.05 vs vehicle.

To determine whether tyrosine phosphorylation of mGluR1a in cerebellar neurons is mediated by SFKs, we investigated the impact of inhibition of SFKs on mGluR1a tyrosine phosphorylation. To inhibit SFKs, we used a widely used SFK inhibitor, PP2 (Hanke et al., 1996). We first evaluated the efficacy and potency of PP2 in inhibiting SFKs in a concentration–response study in rat cerebellar slices. Adding PP2 (0.01, 0.1, 1, and 10 μm, 30 min) greatly reduced the phosphorylation of SFKs at Y416, while PP2 had no effect on a total amount of Fyn or Src (Fig. 5*A*). This effect was clearly concentration dependent. To test the effect of PP2 on specific Fyn, we immunoprecipitated Fyn proteins from the cerebellum after PP2 treatment (Fig. 5*B*). We then analyzed changes in pY416 signals in the immunoprecipitated fraction of Fyn proteins. PP2 (10 μm, 30 min) substantially reduced the pY416 level of Fyn (Fig. 5*C*). PP2, however, did not alter total Fyn expression. These results establish an effective concentration of PP2 in inhibiting Fyn in our brain slice model. We then used this concentration to examine the effect of PP2 on mGluR1a tyrosine phosphorylation.

Adding PP2 to cerebellar slices (10 μm, 30 min) considerably reduced tyrosine phosphorylation of mGluR1a. As shown in Figure 5*D*, mGluR1a immunoreactivity in pY precipitates was largely reduced in PP2-treated slices compared with vehicle control slices. In contrast, PP3, an inactive analog of PP2, did not alter mGluR1a levels in pY precipitates (Fig. 5*E*). Thus, a PP2-sensitive SFK acts as a central kinase responsible for maintaining constitutive tyrosine phosphorylation of mGluR1a in cerebellar neurons.

### Roles of SFKs in regulating surface expression of mGluR1a

**Figure 6.**
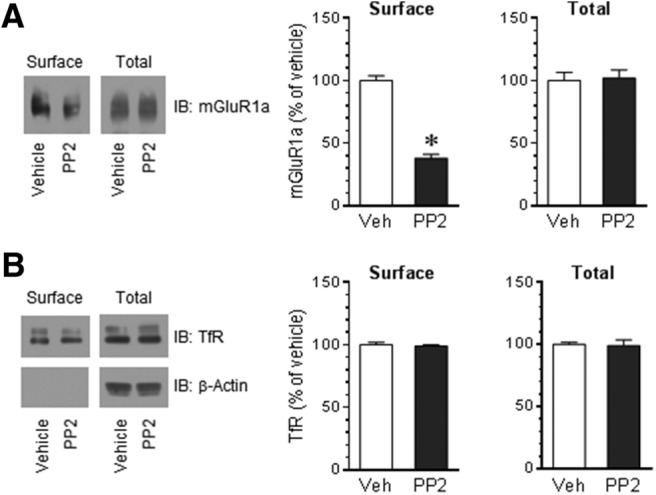
Effects of SFK inhibition on surface expression of mGluR1a in rat cerebellar neurons. ***A***, Effects of the SFK inhibitor PP2 on the surface expression of mGluR1a. Note that PP2 reduced surface levels of mGluR1a, while PP2 did not alter a total amount of mGluR1a. ***B***, Effects of the SFK inhibitor PP2 on the surface expression of TfR. Representative immunoblots are shown to the left of the quantified data. Cerebellar slices were incubated with vehicle (Veh) or PP2 (10 μm, 30 min). Slices were then collected for surface protein biotinylation followed by immunoblot (IB) analysis of the amount of mGluR1a in surface and total protein fractions. Data are presented as the mean ± SEM (*n* = 6 per group) and were analyzed by Student’s *t* test. *p < 0.05 vs vehicle.

Tyrosine phosphorylation of mGluR1a by Fyn may have an impact on surface expression of the receptors. To evaluate this, we investigated whether PP2 alters surface levels of mGluR1a. A surface protein biotinylation protocol was used to monitor changes in the surface expression of mGluR1a (see Materials and Methods). In rat cerebellar slices, the application of PP2 (10 μm, 30 min) markedly reduced the amount of mGluR1a in the surface fraction (Fig. 6*A*). The total cellular levels of mGluR1a were not altered. A surface protein, the TfR, and an intracellular protein, β-actin, were measured as a surface and intracellular protein control, respectively. As shown in Figure 6*B*, TfRs were densely seen in surface and total fractions, whereas β-actin was not shown in surface fractions, demonstrating the selectivity of our biotinylation method in cross-linking surface proteins. No significant change in the surface levels of TfRs was seen following PP2 incubation (Fig. 6*B*). These results indicate a significant role of SFKs in determining the number of mGluR1a in the surface compartment of cerebellar neurons.

### Roles of SFKs in regulating mGluR1-IP_3_ signaling

**Figure 7.**
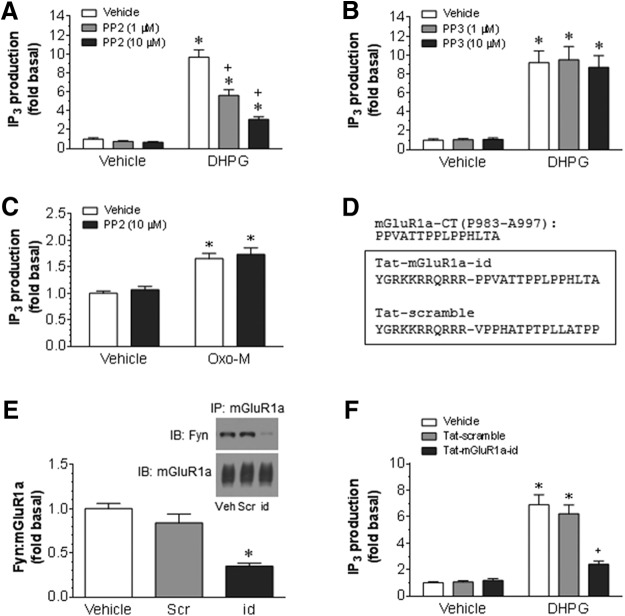
Effects of inhibition of SFKs or disruption of Fyn–mGluR1a interactions on the mGluR1-mediated IP_3_ production in rat cerebellar neurons. ***A***, ***B***, Effects of PP2 (***A***) or PP3 (***B***) on the DHPG-stimulated IP_3_ formation. PP2 or PP3 (1 and 10 μm) was applied 30 min before and during 20 s incubation of DHPG (50 μm). Note that PP2 reduced IP_3_ responses to DHPG, while PP3 did not. Both agents hardly altered basal IP_3_ levels. ***C***, Effects of PP2 on the oxotremorine-M-stimulated IP_3_ formation. PP2 (10 μm) was applied 30 min before and during 20 s incubation of oxotremorine-M (Oxo-M; 10 μm). ***D***, Synthesized Tat fusion peptides. ***E***, Effects of Tat peptides on the interaction between Fyn and mGluR1a. ***F***, Effects of Tat peptides on the DHPG-stimulated IP_3_ formation. Vehicle (Veh), Tat-mGluR1a-id (id), or Tat-scramble (Scr) at 10 μm was applied alone for 45 min (***E***) or 45 min before and during 20 s incubation of 50 μm DHPG (***F***). Note that Tat-mGluR1a-id markedly reduced the interaction of Fyn with mGluR1a (***E***) and the IP_3_ response to DHPG (***F***), while Tat-scramble did not. Experiments were conducted in rat cerebellar slices. Data are presented as the mean ± SEM (*n* = 6/group) and were analyzed by one-way ANOVA. *p < 0.05 vs vehicle (***E***) or vehicle plus vehicle (***A–C***, ***F***). +*p* < 0.05 vs vehicle plus DHPG (***A***, ***F***).

To further explore the functional roles of tyrosine phosphorylation of mGluR1, we investigated the effect of PP2 on the mGluR1-associated signaling activity. Activation of mGluR1 increases phosphoinositide hydrolysis, yielding a key signaling molecule, IP_3_ (Niswender and Conn, 2010; Traynelis et al., 2010). We thus measured the mGluR1-induced IP_3_ yield as function of mGluR1. DHPG, an mGluR1/5 agonist, induced a typical increase in cytosolic IP_3_ levels after it was added to cerebellar slices (50 μm, 20 s; Fig. 7*A*). This increase was completely blocked by an mGluR1-selective antagonist, 3-MATIDA (10 μm; data not shown), verifying the role of mGluR1 in mediating the DHPG-stimulated IP_3_ production. Pretreatment with PP2 (1 µm, 30 min before DHPG) significantly reduced IP_3_ responses to DHPG. A greater reduction in IP_3_ responses was seen following pretreatment with PP2 at a higher concentration (10 µm). In contrast to PP2, PP3 at two concentrations (1 and 10 µm) had no significant influence over IP_3_ responses to DHPG (Fig. 7*B*). Neither PP2 nor PP3 altered basal levels of IP_3_. These results indicate that the inhibition of SFKs leads to a reduction in mGluR1-mediated IP_3_ production.

Muscarinic acetylcholine receptors (mAChRs) are expressed in the cerebellum (Whitham et al., 1991; Tayebati et al., 2001; Billups et al., 2006). Of the five mAChR subtypes, M1, M3, and M5 subtypes are G_αq_-coupled receptors and are linked to the activation of PLCβ1 (Felder, 1995; Wess, 1996). The activation of G_αq_-coupled mAChRs, predominantly M3 receptors, present on cerebellar Purkinje neurons with a mAChR agonist oxotremorine-M blocked long-term potentiation at cerebellar parallel fiber–Purkinje cell synapses (Rinaldo and Hansel, 2013). In this study, we found that oxotrmorine-M (10 μm, 20 s) elevated IP_3_ levels in cerebellar slices (Fig. 7*C*). This elevation was not affected by PP2. Thus, SFK activity is not involved in the regulation of IP_3_ responses to mAChR activation.

We next synthesized a cell-permeable, Tat-fusion peptide that contains the proline-rich motifs (PXXP) in the CT1c fragment (Fig. 7*D*). This peptide was designed to interfere with the Fyn–mGluR1a interaction and was thus named as an interaction-dead (Tat-mGluR1a-id) peptide. In fact, the application of Tat-mGluR1a-id (10 μm, 45 min) significantly reduced the interaction of Fyn with mGluR1a in cerebellar slices, while a sequence-scrambled control peptide (Tat-scramble) did not (Fig. 7*E*). This establishes the importance of these proline-rich motifs for the binding of Fyn to mGluR1a. Of note, Tat-mGluR1a-id reduced the IP_3_ response to DHPG, while Tat-scramble had no effect (Fig. 7*F*). Thus, the Fyn–mGluR1a interaction is critical for the mGluR1a–IP_3_ signaling.

### Fyn regulates mGluR1a in HEK293T cells

**Figure 8.**
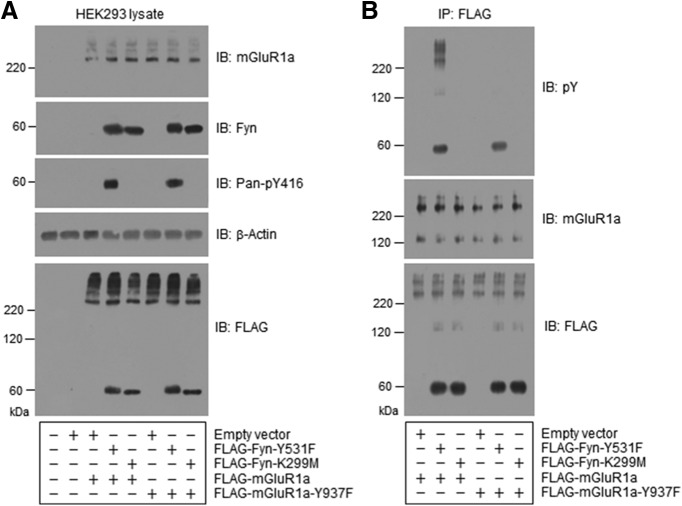
Fyn-induced phosphorylation of mGluR1a at Y937 in HEK293T cells. ***A***, Representative immunoblots illustrating the expression of mGluR1a (WT and Y937F) and Fyn (Y531F and K299M) transfected in HEK293T cells. ***B***, Tyrosine phosphorylation of mGluR1a by cotransfection of active Fyn in HEK293T cells. Note that tyrosine phosphorylation of mGluR1a was seen in HEK293T cells cotransfected with active Fyn (Y531F) but not inactive Fyn (K299M). Such phosphorylation occurred to WT mGluR1a but not to mGluR1a-Y937F. FLAG-tagged mGluR1a (WT and Y937F) and FLAG-tagged Fyn (Y531F and K299M) in pcDNA3.1 + C(K)DYK vectors (empty vectors) were transfected or cotransfected in HEK293T cells. FLAG-tagged proteins were purified via immunoprecipitation (IP) with an anti-FLAG antibody. Transfected and immunopurified proteins were analyzed by immunoblots (IB).

The role of Fyn in phosphorylating and regulating mGluR1a was further analyzed in HEK293T cells. FLAG-tagged mGluR1a (WT or Y937F, a phosphorylation-deficient mutant due to the mutation of tyrosine 937 to phenylalanine) and Fyn (Y531F or K299M) were readily transfected in HEK293T cells (Fig. 8*A*). The amount of total mGluR1a protein (WT or Y937F) seemed to remain stable after cotransfected with either the constitutively active Fyn (Y531F) or inactive Fyn (K299M). The active and inactive states of Fyn-Y531F and Fyn-K299M, respectively, were confirmed by the detection of pY416 signals in Fyn-Y531F but not Fyn-K299M. Remarkably, tyrosine phosphorylation of mGluR1a at Y937 was induced by active Fyn. As shown in Figure 8*B*, pY signals were detected in FLAG-tagged proteins immunopurified by an anti-FLAG antibody when WT mGluR1a was cotransfected with Fyn-Y531F but not Fyn-K299M. These pY signals were lacking in HEK293T cells transfected with the mGluR1a-Y937 phosphorylation-deficient mutant (Y937F). These results reveal that active Fyn phosphorylates mGluR1a at Y937 in a heterologous expression system.

**Figure 9.**
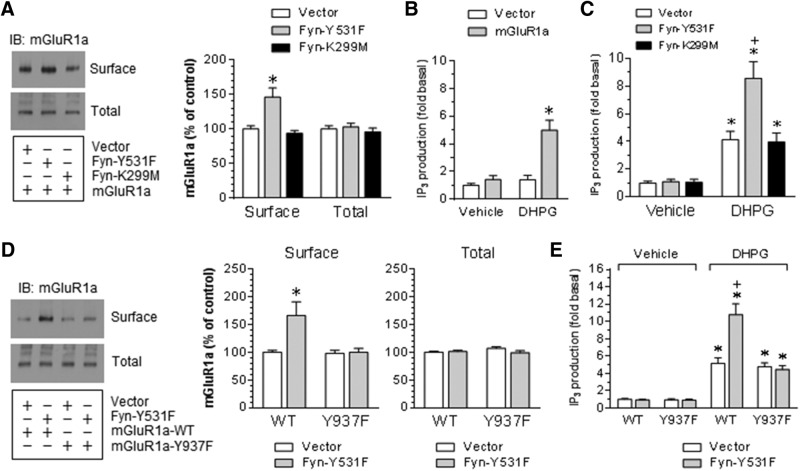
Surface expression of mGluR1a and agonist-stimulated IP_3_ production in HEK293T cells. ***A***, Effects of cotransfection of Fyn on the surface expression of mGluR1a. mGluR1a was cotransfected with active (Y531F) or inactive (K299M) Fyn. Note that surface expression of mGluR1a was elevated by cotransfection with Y531F but not K299M. ***B***, DHPG-stimulated IP_3_ production in HEK293T cells transfected with mGluR1a. ***C***, DHPG-stimulated IP_3_ production in HEK293T cells cotransfected with mGluR1a and active and inactive Fyn. Note that the DHPG-stimulated IP_3_ production was enhanced by cotransfection with Y531F but not K299M. ***D***, Effects of the site-directed mutation at mGluR1a-Y937 on the Fyn-induced surface expression of mGluR1a. mGluR1a WT or Y937 phosphorylation-deficient mutant (Y937F) was cotransfected with active Fyn (Y531F). Note that the mutation of Y937 abolished the active Fyn-induced increase in surface expression of mGluR1a. ***E***, Effects of the site-directed mutation at mGluR1a-Y937 on the Fyn-induced increase in IP_3_ responses to DHPG. Note that cotransfection with active Fyn (Y531F) augmented IP_3_ responses to DHPG in HEK293T cells cotransfected with WT but not Y937F mGluR1a. Representative immunoblots are shown to the left of the quantified data (***A***, ***D***). Proteins were visualized by immunoblots (IB). In IP_3_ assays, DHPG (50 μm) was added and cells were collected 20 s after DHPG incubation. Data are presented as the mean ± SEM (*n* = 3-6/group) and were analyzed by one-way ANOVA. *p < 0.05 vs vector (***A***, ***B***, ***D***) or vector plus vehicle (***C***, ***E***). +*p* < 0.05 vs vector plus DHPG (***C***, ***E***).

To determine the role of Fyn in regulating the surface expression of mGluR1a, we cotransfected active or inactive Fyn with mGluR1a in HEK293T cells. We found that active Fyn (i.e. Y531F) significantly increased mGluR1a surface expression, while inactive Fyn (K299M) did not (Fig. 9*A*). Both active and inactive Fyn did not change the level of total mGluR1a proteins. Thus, in transfected HEK293T cells, Fyn enhances the surface expression of mGluR1a.

We next determined whether Fyn regulates mGluR1a signaling. IP_3_ production was measured as a readout of mGluR1a signaling. DHPG induced an increase in IP_3_ levels in HEK293T cells transfected with mGluR1a, but not with an empty vector (Fig. 9*B*), indicating successful expression of functional mGluR1a in HEK293T cells. When mGluR1a was cotransfected with a Fyn mutant, active Fyn (Y531F) significantly augmented IP_3_ responses to DHPG, while inactive Fyn (K299M) did not (Fig. 9*C*). This indicates that Fyn positively regulates mGluR1a signaling in transfected HEK293T cells.

To examine whether Fyn regulates mGluR1a via phosphorylating Y937, we conducted a site-directed mutation study. A phosphorylation-deficient mutant of mGluR1a (Y937F) was cotransfected with active Fyn (Y531F) in HEK293T cells. The effects of Fyn-Y531F on surface expression and signaling activity of mGluR1a were then examined in transfected HEK293T cells. WT mGluR1a and mGluR1a-Y937F exhibited a similar level of surface and total expression (Fig. 9*D*). However, active Fyn-induced increases in surface expression of mGluR1a were seen only in WT, but not mutant mGluR1a (Y937F), indicating that Y937 phosphorylation is critical for this event. In IP_3_ assays, the Y937F mutation completely abolished an augmentation of IP_3_ responses to DHPG induced by the cotransfection of active Fyn (Fig. 9*E*). These results support that the phosphorylation of mGluR1a at Y937 is essential for Fyn to enhance mGluR1a signaling.

## Discussion

This study was conducted to explore a new substrate of Fyn. We found that an mGluR subtype, mGluR1a, is a target of Fyn. Fyn directly bound to mGluR1a CT *in vitro*. Similarly, endogenous and synapse-enriched Fyn interacted with mGluR1a in rat cerebellar neurons. Active Fyn phosphorylated mGluR1a at a conserved tyrosine site (Y937) in the CT region. This phosphorylation was constitutively active. The phosphorylation at Y937 was an important step for promoting surface expression of mGluR1a. By regulating surface expression of the receptor, Fyn controlled the mGluR1a-associated signaling transduction. In sum, the results obtained *in vitro* and *in vivo* support mGluR1a as a novel synaptic substrate of Fyn. Through directly binding to and phosphorylating mGluR1a, Fyn regulates mGluR1a in surface expression and postreceptor signaling.

The Fyn–mGluR1a interaction is a new model discovered in this study. This interaction is characterized by occurring in mGluR1a CT, a region thought to be a major protein–protein interaction area. Indeed, mGluR1a CT is relatively large and is a binding domain for most mGluR1a-interacting partners so far identified (Enz, 2012; Fagni, 2012). As to the specific binding site in mGluR1a CT, proline-rich PXXP motifs that are preferentially recognized by a protein–protein interaction module (SH3 domain) of Fyn (Ren et al., 1993) exist in the CT1c region. Consistent with this, CT1c but not CT1a and CT1b bound to Fyn. Thus, a PXXP-containing zone in CT1c likely harbors a binding site to Fyn. This is supported by the finding that a PXXP-containing peptide derived from mGluR1a-CT1c reduced the association between Fyn and mGluR1a in cerebellar neurons (this study). In addition to the binding site in the CT region, tyrosine phosphorylation of mGluR1a induced by Fyn occurred at a residue (Y937) within the CT1c. Thus, the CT1c is a major region that Fyn interacts with to tyrosine phosphorylate and regulate the receptor.

Another important characteristic of the Fyn–mGluR1a coupling is its constitutively active nature. Following the demonstration of the binding between recombinant Fyn and mGluR1a proteins and tyrosine phosphorylation of mGluR1a *in vitro*, we investigated the interaction between two native proteins in neurons. In cerebellar neurons expressing Fyn (Umemori et al., 1992; Bare et al., 1993; Cioni et al., 2013) and mGluR1a (Martin et al., 1992; Shigemoto et al., 1993), the Fyn–mGluR1a complex was detected under basal conditions. In parallel with the basal Fyn–mGluR1a interaction, the Fyn-mediated tyrosine phosphorylation of mGluR1a occurred significantly under normal conditions. More importantly, the constitutively active phosphorylation is functionally relevant and contributes to the regulation of surface expression and signaling of mGluR1a under normal conditions (see below).

Fyn is enriched at synaptic sites and is conceived to play a critical role in regulating local synaptic proteins (Ohnishi et al., 2011; Schenone et al., 2011). Several substrates and binding partners of Fyn have been previously discovered at synaptic sites. Central among them is the NMDA glutamate receptor (Suzuki and Okumura-Noji, 1995; Köhr and Seeburg, 1996; Trepanier et al., 2012; Knox and Jiang, 2015). In addition, two prominent synaptic scaffold proteins, PSD-95 and PSD-93, are substrates of Fyn (Nada et al., 2003; Du et al., 2009). This study adds mGluR1a as another synaptic substrate of Fyn. The results from a series of biochemical and functional studies conducted in the present work are consistent with a notion that mGluR1a is subjected to the tyrosine phosphorylation and regulation by Fyn.

Phosphorylation is an important mechanism for the regulation of GPCRs. As a typical GPCR, mGluR1 is subjected to the regulation by a phosphorylation-dependent mechanism. Several common protein kinases have been implicated in this event (Dhami and Ferguson, 2006; Kim et al., 2008; Mao et al., 2008). For example, PKC is involved in the phosphorylation and regulation of mGluR1 based on a number of early studies (Manzoni et al., 1990; Catania et al., 1991; Thomsen et al., 1993; Alaluf et al., 1995; Medler and Bruch, 1999; Francesconi and Duvoisin, 2000). calcium/calmodulin-dependent protein kinase II (CaMKII) is another kinase that contributes to the regulation of mGluR1a. By phosphorylating threonine 871 near the G-protein-coupling domain of mGluR1a (Dhami and Ferguson, 2006), CaMKII serves as an important element in forming a feedback loop that facilitates the agonist-induced desensitization of mGluR1a in striatal neurons (Jin et al., 2013). However, both PKC and CaMKII catalyze phosphorylation at serine and threonine residues. Whether a kinase that phosphorylates proteins at a tyrosine site is involved in the regulation of mGluR1 is unclear. This study provides evidence supporting a new tyrosine kinase–GPCR model that a synapse-enriched tyrosine kinase Fyn acts as a key regulator of mGluR1. However, unlike the activity-dependent and inhibitory nature of the regulation of mGluR1a by PKC and CaMKII, Fyn functions significantly under basal conditions and facilitates the receptors in their expression and postreceptor signaling. More specifically, in cerebellar neurons where mGluR1a and Fyn are predominantly expressed (Umemori et al., 1992; Bare et al., 1993; Fotuhi et al., 1993), Fyn constitutively binds to the largest intracellular domain of mGluR1a, which may serve to accumulate the kinase at synaptic sites under basal conditions. Synaptic Fyn then phosphorylates a tyrosine residue in mGluR1a to promote steady-state surface expression of the receptors and thereby maintain the activity level of mGluR1a signaling. Fyn may regulate trafficking, endocytosis, dimerization, or other steps important for surface expression of mGluR1a to modulate the number of the receptors in the surface compartment, although exact underlying mechanisms are unclear at present. Future studies need to clarify accurate mechanisms underlying the impact of the Fyn phosphorylation of mGluR1a on the surface expression of the receptors.

## Synthesis

Reviewing Editor: Kirill Martemyanov, The Scripps Research Institute Decisions are customarily a result of the Reviewing Editor and the peer reviewers coming together and discussing their recommendations until a consensus is reached. When revisions are invited, a fact-based synthesis statement explaining their decision and outlining what is needed to prepare a revision will be listed below. The following reviewer(s) agreed to reveal their identity: Paul Kammermeier.

Both reviewers agreed that results presented in the manuscript reveal new mechanism for receptor regulation. The findings demonstrating novel interaction of Fyn kinase with mGluR1 and its effects on receptor phosphorylation and trafficking are considered important advance with implications for several areas of neurobiology. However, the reviewers also identified several areas of concerns and it was felt that these issues need to be addressed to increase confidence in the interpretation of the results and enhance impact of the report. Specifically I would like to direct your attention to the following areas that will be critical for you to address upon revision of your manuscript.

1. Performing controls in biochemical experiments: adding loading controls, performing GST carrier only pull-downs, and showing inputs.

2. It would be important to demonstrate if Fyn is forming a direct complex with mGluR1 in vivo and that this interaction results in the receptor phosphorylation. For example, mGluR1 phosphorylation in immunoprecipitation experiments will need to be looked at and co-immunoprecipitations need to be carried out in detergent solubilized preparations rather than intact membrane fractions.

3. It would be important to carefully revise statistical methods indicating for each experiment (either in the text or figure legends, the number of biological replicates, times an experiment was repeated, and statistical test used).

4. Whenever possible, provide quantitative information, calculating phosphorylation stoichiometry, fraction of proteins detected in the complex, etc.

5. Performing additional experiments to (i) further delineate the binding site of Fyn in mGluR1a C-tail, (ii) block interaction of Fyn with mgluR1a using interfering peptides, and testing effects of PP2 inhibition on another Gq-coupled receptor.

6. Revise the discussion making it more to the point and directly related to the effects reported in a context of GPCR signaling mechanisms.

7. Addressing typos and imprecise use of terminology when referring to pharmacological effects (i.e. efficacy/potency)

For your reference, I am providing unedited comments of both reviewers below. These should provide specific guidance for correcting the major issues indicated above as well as for addressing minor points to improve the manuscript.

Specific Reviewer's Comments

Reviewer #1

The manuscript “An essential role of Fyn in the modulation of metabotropic glutamate receptor 1 in cerebellar neurons” describes a study in which a novel interaction between mGluR1 and Fyn is described. Authors demonstrate that the interaction leads to tyrosine phosphorylation of the C-tail of mGluR1 and narrowed the interaction site to a portion of the tail, and finally identified the phosphorylation site to Y937. Finally, they showed that this resulted in increased membrane expression of mGluR1 and consequent enhanced signaling. Experiments showed that the enhanced membrane expression of mGluR1 was a direct result of Y937 phos. The experiments described were thorough and well described. Only a few minor issues should be addressed:

1- Line 202: authors state “we synthesized two recombinant proteins...” but describe THREE in the figure.

2- The section starting “The cerebellum was used in pull-down...” on lines 243-248 (pp. 11-12) appears to be introducing experiments described in Fig. 4 rather than Fig. 3. This should be moved to the next section.

3- line 288-289 “We first evaluated the efficacy of PP2...” should read “efficacy and potency...” since they did perform a concentration-response curve.

4- A related issue, the authors use the term Efficacy several times to describe the effects of Fyn on mGluR1 signaling ("increased efficacy", for example). In this case, the use of this term is not appropriate since they only examined a single DHPG concentration. This enhanced signaling could result from either increased efficacy or potency. The term should be changed to something less specific, such as “enhanced signaling".

5- In Fig. 8, it is difficult to discern which constructs are FLAG tagged in the illustrated experiments. This figure should be labeled better.

Reviewer #2

The manuscript eN-NWR-0096-17 investigates the regulation of mGluR1a signaling by the src kinase Fyn. The authors provide evidence that the kinase binds to the receptor and constitutively tyrosine phosphorylates the receptor on tyr 937. The authors also suggest that Fyn can be co-immunoprecipitated from cerebellar synaptosomes with mGluR1a and that mGluR1a can be similarly be co-immunoprecipitated with Fyn or pY antibody. In addition, they provide evidence that Fyn contributes to the cell surface retention of mGluR1 and that tyrosine phosphorylation is required for agonist-stimulated but not basal mGluR1-dependent IP formation.

The manuscript puts forth a novel hypothesis that constitutive tyrosine phosphorylation of mGluR1a by Fyn contributes to the regulation of mGluR1a coupling to IP formation. However, a number of issues still need to be addressed both experimentally and in the form of presentation of the manuscript.

1. In the GST protein phosphorylation and pull down experiments described in Figure 1 - 3, there is no indication in the text or the Figure legends of the amount of fusion protein utilized in any of the experiments, nor are there any GST-fusion loading controls provide (this is essential). Furthermore, in Figure 1 although there is evidence of tyrosine phosphorylation by autoradiography, the stoichiometry of the phosphorylation of the GST-fusion protein has not been determined. This information could be easily provided given that the authors are working with purified proteins and is essential to determine whether the extent of tyrosine phosphorylation is functionally relevant. Also in Figure 1D-F what is the nature of the multiple phosphorylated bands show in the autoradiograph. In Figure 2D, despite the authors' conclusion that only Y937 is phosphorylated, phosphorylation is reduced for the Y955F mutant and the lower phosphorylated band is nearly completely lost. The authors need to provide an explanation for this. There is also no indication of how many times the experiments in Figures 1 and 2 have been performed.

2. The authors conclude that Fyn interacts with the receptor via polyproline rich motifs in the mGluR1a C-tail. This needs to be confirmed experimentally.

3. The authors utilize an unorthodox approach for pull down experiments from cerebellar tissue in Figure 4 and 5 in which they immunoprecipitate the receptor, Fyn and phosphotyrosinated proteins from a P2 (synaptosomal fraction). Previous studies utilizing a similar approach to immunoprecipitate the NMDAR receptor identified a protein complex that included more than 100 protein along with mGluR1 (PMID: 10707975). A blot showing tyrosine phosphorylation of mGluR1a directly immunoprecipitated with a mGluR1 antibody should be provided as the data provided in Figure 4D is less than convincing. Moreover, the co-immunoprecipitation of Fyn and mGluR1a should be determined from a detergent solubilized preparation. Loading controls are also missing for Figure 5C-E.

4. In Figure 7 the authors need to provide additional data showing that the effects of the PP2 inhibitor are selective to mGluR1a signaling by investigating the effects of the PP2 inhibitor on the signaling of another Gq-coupled receptor. Independent validation that this result is the consequence of Fyn interactions with mGluR1a should be provided using a cell permeant interference peptide.

5. In Figure 8, why does the Flag antibody pick up a 60 kDa band in addition to mGluR1a what I can only assume this to be Flag-tagged Fyn in the Flag immunoblot? If so, why the same tag on both proteins? Loading controls are also missing for this experiment and it is not clear whether the blots provided are indicative of a single experiment.

6. The discussion of the manuscript is terse, short and often irrelevant to the results presented. The authors need to provide some discussion of how there results fits into the overall understanding of the regulation of receptor signaling, why its unique and propose a mechanism by which tyrosine phosphorylation might prevent G protein coupling.

## References

[B1] Alaluf S, Mulvihill ER, McIlhinney RA (1995) Rapid agonist mediated phosphorylation of the metabotropic glutamate receptor 1α by protein kinase C in permanently transfected BHK cells. FEBS Lett 367:301–305. 10.1016/0014-5793(95)00575-T 7607328

[B2] Bare DJ, Lauder JM, Wilkie MB, Maness PF (1993) p59fyn in rat brain is localized in developing axonal tracts and subpopulations of adult neurons and glia. Oncogene 8:1429–1436. 8502471

[B3] Billups D, Billups B, Challiss RA, Nahorski SR (2006) Modulation of Gq-protein-coupled inositol trisphosphate and Ca^2+^ signaling by the membrane potential. J Neurosci 26:9983–9995. 10.1523/JNEUROSCI.2773-06.2006 17005862 PMC2266565

[B4] Burgaya F, Menegon A, Menegoz M, Valtorta F, Girault JA (1995) Focal adhesion kinase in rat central nervous system. Eur J Neurosci 7:1810–1821. 7582133 10.1111/j.1460-9568.1995.tb00700.x

[B5] Cansev M, Orhan F, Yaylagul EO, Isik E, Turkyilmaz M, Aydin S, Gumus A, Sevinc C, Coskun N, Ulus IH, Wurtman RJ (2015) Evidence for the existence of pyrimidinergic transmission in rat brain. Neuropharmacology 91:77–86. 10.1016/j.neuropharm.2014.12.019 25541414

[B6] Catania MV, Aronica E, Sortino MA, Canonico PL, Nicoletti F (1991) Desensitization of metabotropic glutamate receptors in neuronal cultures. J Neurochem 56:1329–1335. 1672146 10.1111/j.1471-4159.1991.tb11429.x

[B7] Cheng SH, Espino PC, Marshall J, Harvey R, Merrill J, Smith AE (1991) Structural elements that regulate pp59c-fyn catalytic activity, transforming potential, and ability to associate with polyomavirus middle-T antigen. J Virol 65:170–179. 1985196 10.1128/jvi.65.1.170-179.1991 PMC240502

[B8] Cioni JM, Telley L, Saywell V, Cadilhac C, Jourdan C, Huber AB, Huang JZ, Jahannault-Talignani C, Ango F (2013) SEMA3A signaling controls layer-specific interneuron branching in the cerebellum. Curr Biol 23:850–861. 10.1016/j.cub.2013.04.007 23602477

[B9] Cooke MP, Perlmutter RM (1989) Expression of a novel form of the fyn proto-oncogene in hematopoietic cells. New Biol 1:66–74. 2488273

[B10] Dhami GK, Ferguson SS (2006) Regulation of metabotropic glutamate receptor signaling, desensitization and endocytosis. Pharmacol Ther 111:260–271. 10.1016/j.pharmthera.2005.01.008 16574233

[B11] Du CP, Gao J, Tai JM, Liu Y, Qi J, Wang W, Hou XY (2009) Increased tyrosine phosphorylation of PSD-95 by Src family kinase after brain ischaemia. Biochem J 417:277–285. 10.1042/BJ20080004 18721130

[B12] Edbauer D, Cheng D, Batterton MN, Wang CF, Duong DM, Yaffe MB, Peng J, Sheng M (2009) Identification and characterization of neuronal mitogen-activated protein kinase substrates using a specific phosphomotif antibody. Mol Cell Proteomics 8:681–695. 10.1074/mcp.M800233-MCP200 19054758 PMC2667352

[B13] Enz R (2007) The trick of the tail: protein-protein interactions of metabotropic glutamate receptors. Bioessays 29:60–73. 10.1002/bies.20518 17187376

[B14] Enz R (2012) Metabotropic glutamate receptors and interacting proteins: evolving drug targets. Curr Drug Targets 13:145–156. 21777188 10.2174/138945012798868452

[B15] Fagni L (2012) Diversity of metabotropic glutamate receptor-interacting proteins and pathophysiological functions. Adv Exp Med Biol 970:63–79. 10.1007/978-3-7091-0932-8_3 22351051

[B16] Felder CC (1995) Muscarinic acetylcholine receptors: signal transduction through multiple effectors. FASEB J 9:619–625. 7768353

[B17] Fotuhi M, Sharp AH, Glatt CE, Hwang PM, von Krosigk M, Snyder SH, Dawson TM (1993) Differential localization of phosphoinositide-linked metabotropic glutamate receptor (mGluR1) and the inositol 1,4,5-trisphosphate receptor in rat brain. J Neurosci 13:2001–2012. 8386753 10.1523/JNEUROSCI.13-05-02001.1993 PMC6576569

[B18] Francesconi A, Duvoisin RM (2000) Opposing effects of protein kinase C and protein kinase A on metabotropic glutamate receptor signaling: selective desensitization of the inositol trisphosphate/Ca^2+^ pathway by phosphorylation of the receptor-G protein-coupling domain. Proc Natl Acad Sci U S A 97:6185–6190. 10.1073/pnas.97.11.6185 10823959 PMC18579

[B19] Guo ML, Fibuch EE, Liu XY, Choe ES, Buch S, Mao LM, Wang JQ (2010) CaMKIIalpha interacts with M4 muscarinic receptors to control receptor and psychomotor function. EMBO J 29:2070–2081. 10.1038/emboj.2010.93 20461055 PMC2892372

[B20] Hanke JH, Gardner JP, Dow RL, Changelian PS, Brissette WH, Weringer EJ, Pollok BA, Connelly PA (1996) Discovery of a novel, potent, and Src family-selective tyrosine kinase inhibitor. Study of Lck- and FynT-dependent T cell activation. J Biol Chem 271:695–701. 8557675 10.1074/jbc.271.2.695

[B21] Hauck CR, Hunter T, Schlaepfer DD (2001) The v-Src SH3 domain facilitates a cell adhesion-independent association with focal adhesion kinase. J Biol Chem 276:17653–17662. 10.1074/jbc.M009329200 11278488

[B22] Hayashi T, Huganir RL (2004) Tyrosine phosphorylation and regulation of the AMPA receptor by Src family tyrosine kinases. J Neurosci 24:6152–6160. 10.1523/JNEUROSCI.0799-04.2004 15240807 PMC6729658

[B23] Jin DZ, Guo ML, Xue B, Fibuch EE, Choe ES, Mao LM, Wang JQ (2013) Phosphorylation and feedback regulation of metabotropic glutamate receptor 1 by calcium/calmodulin-dependent protein kinase II. J Neurosci 33:3402–3412. 10.1523/JNEUROSCI.3192-12.2013 23426668 PMC3711744

[B24] Kato AS, Knierman MD, Siuda ER, Isaac JT, Nisenbaum ES, Bredt DS (2012) Glutamate receptor δ2 associates with metabotropic glutamate receptor 1 (mGluR1), protein kinase Cγ, and canonical transient receptor potential 3 and regulates mGluR1-mediated synaptic transmission in cerebellar Purkinje neurons. J Neurosci 32:15296–15308. 10.1523/JNEUROSCI.0705-12.2012 23115168 PMC6621574

[B25] Kim CH, Lee J, Lee JY, Roche KW (2008) Metabotropic glutamate receptors: phosphorylation and receptor signaling. J Neurosci Res 86:1–10. 10.1002/jnr.21437 17663464

[B26] Knackstedt LA, Trantham-Davidson HL, Schwendt M (2013) The role of ventral and dorsal striatum mGluR5 in relapse to cocaine-seeking and extinction learning. Addict Biol 19:87–101. 10.1111/adb.12061 23710649 PMC3762937

[B27] Knox R, Jiang X (2015) Fyn in neurodevelopment and ischemic brain injury. Dev Neurosci 37:311–320. 10.1159/000369995 25720756 PMC4713834

[B28] Köhr G, Seeburg PH (1996) Subtype-specific regulation of recombinant NMDA receptor-channels by protein tyrosine kinases of the src family. J Physiol 492:445–452. 10.1113/jphysiol.1996.sp021320 9019541 PMC1158839

[B29] Kuwajima M, Hall RA, Aiba A, Smith Y (2004) Subcellular and subsynaptic localization of group I metabotropic glutamate receptors in the monkey subthalamic nucleus. J Comp Neurol 474:589–602. 10.1002/cne.20158 15174075

[B30] Liu XY, Mao LM, Zhang GC, Papasian CJ, Fibuch EE, Lan HX, Zhou HF, Xu M, Wang JQ (2009) Activity-dependent modulation of limbic dopamine D3 receptors by CaMKII. Neuron 61:425–438. 10.1016/j.neuron.2008.12.015 19217379 PMC2650276

[B31] Lujan R, Nusser Z, Roberts JD, Shigemoto R, Somogyi P (1996) Perisynaptic location of metabotropic glutamate receptors mGluR1 and mGluR5 on dendrites and dendritic spines in the rat hippocampus. Eur J Neurosci 8:1488–1500. 8758956 10.1111/j.1460-9568.1996.tb01611.x

[B32] Manzoni OJ, Finiels-Marlier F, Sassetti I, Blockaert J, le Peuch C, Sladeczek FA (1990) The glutamate receptor of the Qp-type activates protein kinase C and is regulated by protein kinase C. Neurosci Lett 109:146–151. 2156190 10.1016/0304-3940(90)90553-l

[B33] Mao LM, Wang JQ (2016) Tyrosine phosphorylation of glutamate receptors by non-receptor tyrosine kinases: roles in depression-like behavior. Neurotransmitter 3:e1118 26942227 PMC4771189

[B34] Mao LM, Liu XY, Zhang GC, Chu XP, Fibuch EE, Wang LS, Liu Z, Wang JQ (2008) Phosphorylation of group I metabotropic glutamate receptors (mGluR1/5) *in vitro* and *in vivo* . Neuropharmacology 55:403–408. 10.1016/j.neuropharm.2008.05.034 18585398 PMC2566896

[B35] Mao LM, Guo ML, Jin DZ, Fibuch EE, Choe ES, Wang JQ (2011) Posttranslational modification biology of glutamate receptors and drug addiction. Front Neuroanat 5:19 10.3389/fnana.2011.00019 21441996 PMC3062099

[B36] Martin LJ, Blackstone CD, Huganir RL, Price DL (1992) Cellular localization of a metabotropic glutamate receptor in rat brain. Neuron 9:259–270. 1323311 10.1016/0896-6273(92)90165-a

[B37] Medler KF, Bruch RC (1999) Protein kinase Cbeta and delta selectively phosphorylate odorant and metabotropic glutamate receptors. Chem Senses 24:295–299. 10400448 10.1093/chemse/24.3.295

[B38] Menegon A, Burgaya F, Baudot P, Dunlap DD, Girault JA, Valtorta F (1999) FAK^+^ and PYK2/CAKβ, two related tyrosine kinases highly expressed in the central nervous system: similarities and differences in the expression pattern. Eur J Neurosci 11:3777–3788. 10.1046/j.1460-9568.1999.00798.x 10583467

[B39] Nada S, Shima T, Yanai H, Husi H, Grant SG, Okada M, Akiyama T (2003) Identification of PSD-93 as a substrate of the Src family tyrosine kinase Fyn. J Biol Chem 278:47610–47621. 10.1074/jbc.M303873200 13129934

[B40] Neet K, Hunter T (1996) Vertebrate non-receptor protein-tyrosine kinase families. Genes Cell 1:147–169. 9140060 10.1046/j.1365-2443.1996.d01-234.x

[B41] Nicoletti F, Bockaert J, Collingridge GL, Conn PJ, Ferraguti F, Schoepp DD, Wroblewski JT, Pin JP (2011) Metabotropic glutamate receptors: from the workbench to the bedside. Neuropharmacology 60:1017–1041. 10.1016/j.neuropharm.2010.10.022 21036182 PMC3787883

[B42] Niswender CM, Conn PJ (2010) Metabotropic glutamate receptors: physiology, pharmacology, and disease. Annu Rev Pharmacol Toxicol 50:295–322. 10.1146/annurev.pharmtox.011008.145533 20055706 PMC2904507

[B43] Ohnishi H, Murata Y, Okazawa H, Matozaki T (2011) Src family kinases: modulators of neurotransmitter receptor function and behavior. Trends Neurosci 34:629–637. 10.1016/j.tins.2011.09.005 22051158

[B44] Okada M (2012) Regulation of the Src family kinase by Csk. Int J Biol Sci 8:1385–1397. 10.7150/ijbs.5141 23139636 PMC3492796

[B45] Orlando LR, Ayala R, Kett LR, Curley AA, Duffner J, Bragg DC, Tsai LH, Dunah AW, Young AB (2009) Phosphorylation of the homer-binding domain of group I metabotropic glutamate receptors by cyclin-dependent kinase 5. J Neurochem 110:557–569. 10.1111/j.1471-4159.2009.06139.x 19457112

[B46] Pabba M, Wong AYC, Ahlskog N, Hristova E, Biscaro D, Nassrallah W, Ngsee JK, Snyder M, Beique JC, Bergeron R (2014) NMDA receptors are upregulated and trafficked to the plasma membrane after sigma-1 receptor activation in the rat hippocampus. J Neurosci 34:11325–11338. 10.1523/JNEUROSCI.0458-14.2014 25143613 PMC6615506

[B47] Panetti TS (2002) Tyrosine phosphorylation of paxillin, FAK, and p130CAS: effects on cell spreading and migration. Front Biosci 7:d143–d150. 11779709 10.2741/A771

[B48] Ren R, Mayer BJ, Cicchetti P, Baltimore D (1993) Identification of a ten-amino acid proline-rich SH3 binding site. Science 259:1157–1161. 8438166 10.1126/science.8438166

[B49] Rinaldo L, Hansel C (2013) Muscarinic acetylcholine receptor activation blocks long-term potentiation at cerebellar parallel fiber-Purkinje cells synapses via cannabinoid signaling. Proc Natl Acad Sci U S A 110:11181–11186. 10.1073/pnas.1221803110 23776234 PMC3704018

[B50] Roskoski R Jr (2005) Src kinase regulation by phosphorylation and dephosphorylation. Biochem Biophys Res Commun 331:1–14. 10.1016/j.bbrc.2005.03.012 15845350

[B51] Saito YD, Jensen AR, Salgia R, Posadas EM (2010) Fyn: a novel molecular target in cancer. Cancer 116:1629–1637. 10.1002/cncr.24879 20151426 PMC2847065

[B52] Schenone S, Brullo C, Musumeci F, Biava M, Falchi F, Botta M (2011) Fyn kinase in brain diseases and cancer: the search for inhibitors. Curr Med Chem 18:2921–2942. 21651487 10.2174/092986711796150531

[B53] Shigemoto R, Nomura S, Ohishi H, Sugihara H, Nakanishi S, Mizuno N (1993) Immunohistochemical localization of a metabotropic glutamate receptor, mGluR5, in the rat brain. Neurosci Lett 163:53–57. 8295733 10.1016/0304-3940(93)90227-c

[B54] Suzuki T, Okumura-Noji K (1995) NMDA receptor subunits epsilon 1 (NR2A) and epsilon 2 (NR2B) are substrates for Fyn in the postsynaptic density fraction isolated from the rat brain. Biochem Biophys Res Commun 216:582–588. 7488151 10.1006/bbrc.1995.2662

[B55] Tabatadze N, Huang G, May RM, Jain A, Woolley CS (2015) Sex differences in molecular signaling at inhibitory synapses in the hippocampus. J Neurosci 35:11252–11265. 26269634 10.1523/JNEUROSCI.1067-15.2015 PMC4532757

[B56] Tayebati SK, Vitali D, Scordella S, Amenta F (2001) Muscarinic cholinergic receptors subtypes in rat cerebellar cortex: light microscope autoradiography of age-related changes. Brain Res 889:256–259. 11166715 10.1016/s0006-8993(00)03146-2

[B57] Thomsen C, Mulvihill ER, Haldeman B, Pickering DS, Hampson DR, Suzdak PD (1993) A pharmacological characterization of the mGluR1α subtype of the metabotropic glutamate receptor expressed in a cloned baby hamster kidney cell line. Brain Res 619:22–28. 10.1016/0006-8993(93)91592-G 7690672

[B58] Traynelis SF, Wollmuth LP, McBain CJ, Menniti ES, Vance KM, Ogden KK, Hansen KB, Yuan H, Myers SJ, Dingledine R (2010) Glutamate receptor ion channels: structure, regulation, and function. Pharmacol Rev 62:405–496. 10.1124/pr.109.002451 20716669 PMC2964903

[B59] Trepanier CH, Jackson MF, MacDonald JF (2012) Regulation of NMDA receptors by the tyrosine kinase Fyn. FEBS J 279:12–19. 10.1111/j.1742-4658.2011.08391.x 21985328

[B60] Twamley GM, Kypta RM, Hall B, Courtneidge SA (1992) Association of Fyn with the activated platelet-derived growth factor receptor: requirements for binding and phosphorylation. Oncogene 7:1893–1901. 1408131

[B61] Twamley-Stein GM, Pepperkok R, Ansorge W, Courtneidge SA (1993) The Src family tyrosine kinases are required for platelet-derived growth factor-mediated signal transduction in NIH 3T3 cells. Proc Natl Acad Sci U S A 90:7696–7700. 8356071 10.1073/pnas.90.16.7696 PMC47209

[B62] Umemori H, Wanaka A, Kato H, Takeuchi M, Tohyama M, Yamamoto T (1992) Specific expression of Fyn and Lyn, lymphocyte antigen receptor-associated tyrosine, in the central nervous system. Mol Brain Res 16:303–310. 1337939 10.1016/0169-328x(92)90239-8

[B63] Wess J (1996) Molecular biology of muscarinic acetylcholine receptors. Crit Rev Neurobiol 10:69–99. 8853955 10.1615/critrevneurobiol.v10.i1.40

[B64] Whitham EM, Challiss RA, Nahorski SR (1991) M3 muscarinic cholinoceptors are linked to phosphoinositide metabolism in rat cerebellar granule cells. Eur J Pharmacol 206:181–189. 1649760 10.1016/s0922-4106(05)80017-3

